# Germline *APOBEC3B* deletion is associated with breast cancer risk in an Asian multi-ethnic cohort and with immune cell presentation

**DOI:** 10.1186/s13058-016-0717-1

**Published:** 2016-05-27

**Authors:** Wei Xiong Wen, Jaslyn Sian-Siu Soo, Pui Yoke Kwan, Elaine Hong, Tsung Fei Khang, Shivaani Mariapun, Christine Shu-Mei Lee, Siti Norhidayu Hasan, Pathmanathan Rajadurai, Cheng Har Yip, Nur Aishah Mohd Taib, Soo Hwang Teo

**Affiliations:** Cancer Research Malaysia, Subang Jaya, Selangor Malaysia; Breast Cancer Research Unit, University Malaya Cancer Research Institute, Faculty of Medicine, University Malaya, Kuala Lumpur, Malaysia; Institute of Mathematical Sciences, Faculty of Science, University Malaya, Kuala Lumpur, Malaysia; Sime Darby Medical Centre, Subang Jaya, Selangor Malaysia; Department of Surgery, Faculty of Medicine, University Malaya Medical Centre, Kuala Lumpur, Malaysia

**Keywords:** APOBEC3B, Asian, Breast cancer, Breast cancer risk, Immune response

## Abstract

**Background:**

APOBEC3B is a cytosine deaminase implicated in immune response to viral infection, cancer predisposition and carcinogenesis. Germline *APOBEC3B* deletion is more common in East Asian women and confers a modest risk to breast cancer in both East Asian and Caucasian women. Analysis of tumour samples from women of European descent has shown that germline *APOBEC3B* deletion is associated with an increased propensity to develop somatic mutations and with an enrichment for immune response-related gene sets. However, this has not been examined in Asian tumour samples, where population differences in genetic and dietary factors may have an impact on the immune system.

**Methods:**

In this study, we determined the prevalence of germline *APOBEC3B* deletion and its association with breast cancer risk in a cross-sectional hospital-based Asian multi-ethnic cohort of 1451 cases and 1442 controls from Malaysia. We compared gene expression profiles of breast cancers arising from *APOBEC3B* deletion carriers and non-carriers using microarray analyses. Finally, we characterised the overall abundance of tumour-infiltrating immune cells in breast cancers from TCGA and METABRIC using ESTIMATE and relative frequency of 22 immune cell subsets in breast cancers from METABRIC using CIBERSORT.

**Results:**

The minor allelic frequency of *APOBEC3B* deletion was estimated to be 0.35, 0.42 and 0.16 in female populations of Chinese, Malay and Indian descent, respectively, and that germline *APOBEC3B* deletion was associated with breast cancer risk with odds ratios of 1.23 (95 % CI: [1.05, 1.44]) for one-copy deletion and 1.38 (95 % CI: [1.10, 1.74]) for two-copy deletion compared to women with no deletion. Germline *APOBEC3B* deletion was not associated with any clinicopathologic features or the expression of any *APOBEC* family members but was associated with immune response-related gene sets (FDR *q* values < 0.05). Analysis of breast cancers from METABRIC revealed breast cancers from *APOBEC3B* deletion carriers to have significantly higher abundance of tumour-infiltrating immune cells (*P* < 0.001).

**Conclusions:**

Taken together, our data suggests that tumour-infiltrating immune cells may be an important feature of breast cancers arising in women with *APOBEC3B* germline deletion, and that this may be of particular interest in Asian women where the germline deletion is more common.

**Electronic supplementary material:**

The online version of this article (doi:10.1186/s13058-016-0717-1) contains supplementary material, which is available to authorized users.

## Background

Apolipoprotein B mRNA-editing enzyme, catalytic polypeptide-like 3B (APOBEC3B) is a member of the APOBEC family of cystidine deaminases whose canonical function is in innate immune defence [1]. APOBEC3B plays role in retrovirus and endogenous retrotransposon restriction by hyperediting complementary DNA (cDNA) intermediates [1]. Recent studies on whole genome- and exome-sequenced cancers have implicated the enzymatic activity of the APOBEC family in two of the 21 mutational signatures, suggesting that APOBEC mutational processes may be a key driver to carcinogenesis [2–4]. Signature 2 consists of predominantly C to T transitions in the TCX sequence context while Signature 13 consists of predominantly C to G transversions in the TCX sequence context [2]. Given that *APOBEC3B* expression is upregulated in breast tumours, and that its upregulation is associated with APOBEC-associated mutational signatures [3–5], it has been suggested that APOBEC3B may be an endogenous source of mutations in cancers, especially breast cancer.

The *APOBEC3B* deletion allele frequency is estimated to be 37 % in East Asian populations, and 6 % in Europeans [6]. The deletion involves the removal of a sequence of length 29.5 kb between apolipoprotein B mRNA-editing enzyme, catalytic polypeptide-like 3A (*APOBEC3A*) and *APOBEC3B*, resulting in a hybrid transcript consisting of the coding region from *APOBEC3A* and the 3′UTR from *APOBEC3B. APOBEC3B* deletion polymorphism is associated with increased risk to breast cancer, with odds ratios (ORs) of 1.31 (95 % confidence interval [Cl]: [1.21, 1.42]) for one-copy deletion and 1.76 (95 % Cl: [1.57, 1.97]) for two-copy deletion in East Asian women [7], and 1.21 (95 % Cl: [1.02, 1.43]) for one-copy deletion and 2.29 (95 % Cl: [1.04, 5.06]) for two-copy deletion in women of European descent [8]. The prevalence of germline *APOBEC3B* deletion and its association with breast cancer remain unknown in other populations.

Given that APOBEC3B has been proposed to be an endogenous source of mutations, it is paradoxical that germline loss of the *APOBEC3B* results in increased propensity to develop tumours with hypermutation. Analysis of breast cancers from The Cancer Genome Atlas (TCGA) showed that germline *APOBEC3B* deletion is associated with an OR of 2.68 for one-copy deletion, and an OR of 3.82 for two-copy deletion with tumours that have a hypermutator phenotype [9]. One plausible explanation is that germline loss of *APOBEC3B* results in hypermutation driven by other endogenous sources of mutation, for example, by other members of the APOBEC cytosine deaminases. Furthermore, it has been proposed that hypermutation in the absence of *APOBEC3B* generates tumour-specific antigens which activate the immune system. Indeed, immune response-related gene sets were found to be enriched in breast cancers arising from *APOBEC3B* deletion carriers [10].

The purpose of this study is to determine the prevalence of germline *APOBEC3B* deletion and its association with breast cancer risk in a multi-ethnic Asian cohort of Chinese, Malay and Indian women in Malaysia, to compare the gene expression profiles of breast cancers arising from Asian *APOBEC3B* deletion carriers and non-carriers, and lastly, to characterise the presentation of tumour-infiltrating immune cells in breast cancers.

## Methods

### Study subjects

All breast cancer patients who participated in the Malaysian Breast Cancer Genetic Study between October 2002 and March 2014 were included in a hospital-based cohort of breast cancer cases. The cases were drawn from two hospitals, namely University Malaya Medical Centre and Sime Darby Medical Centre [11]. Of the 2452 breast cancer patients, 378 individuals were excluded from the study if they were either (a) male breast cancer patients (*n* = 7); (b) diagnosed with non-invasive breast cancer (*n* = 133); (c) had missing histopathology data (*n* = 151); (d) had insufficient or low-quality genomic DNA samples (*n* = 46) or (e) were of ethnicity other than Chinese, Malay or Indian, or unknown ethnicity (*n* = 58). Control subjects included women who attended an opportunistic mammography screening programme at Sime Darby Medical Centre between October 2011 and September 2014 [11]. Of the 1819 control subjects, 271 individuals were excluded from the study if they were either (a) diagnosed with invasive breast cancer (*n* = 12); (b) were younger relatives of study participants (*n* = 209) or (c) were of ethnicity other than Chinese, Malay or Indian, or unknown ethnicity (*n* = 64). The remaining cases and controls were frequency-matched on 5-year age group within each ethnic group. Thus, a total of 1468 breast cancer cases consisting of 991 Chinese, 253 Indians, and 224 Malays and a total of 1451 healthy controls consisting of 986 Chinese, 246 Indians and 219 Malays were included in the study. All study participants provided written informed consent. The study was approved by the Medical Ethics Committee of University Malaya Medical Centre (application number: 842.9) and the Independent Ethics Committee of Sime Darby Medical Centre (application numbers: 201109.4 and 201208.1).

### Copy number analyses

Primers and probes highly specific to the target gene, *APOBEC3B* (assay ID: Hs04504055_cn), and the reference gene, *RNase P*, were purchased from Applied Biosystems (Foster City, CA, USA). Duplex real-time quantitative PCR assays were performed on 384-well plates using Applied Biosystems ViiA 7 Real-Time PCR System (Applied Biosystems, Foster City, CA, USA) according to the manufacturer’s instructions. Each plate included one negative control (water) and one calibrator. The calibrator is a sample previously confirmed to carry two copies of *APOBEC3B* using droplet digital PCR. All samples were tested in triplicate, and fluorescence signals were normalised to the passive reference dye ROX. The quantitative PCR amplification curves were analysed using ViiA 7 software on a plate-by-plate basis. CopyCaller software version 2.0 (Applied Biosystems, Foster City, CA, USA) was used to assign the copy number from the raw quantification cycle (Cq). Samples with confidence values below 95 % or absolute *z* scores of 2.65 or greater were excluded from further analysis. A total of 1451 breast cancer cases consisting of 984 Chinese, 245 Indians, and 222 Malays and a total of 1442 healthy controls consisting of 985 Chinese, 239 Indians and 218 Malays were included in the analysis.

### Microarray experiment

Twenty-eight invasive breast cancers which were positive for expression of estrogen receptor (ER) and negative for expression of human epithelial receptor 2 (HER2) (by immunohistochemistry) were selected for gene expression analysis. The clinicopathologic features of the tumour samples are shown in Additional file 1: Table S1. The samples were frequency- and ethnicity-matched between each genotype group. Hematoxylin and eosin-stained frozen sections were obtained and tumour content was confirmed by a pathologist (PR). RNA from each sample (50 ng) was used to generate biotinylated sense-strand cDNA using the GeneChip WT Plus Reagent Kit according to the manufacturer’s instruction (Affymetrix Inc., Santa Clara, CA, USA). Microarray experiments were performed using GeneChip Human Transcriptome Array 2.0 (Affymetrix Inc., Santa Clara, CA, USA) according to the manufacturer’s instructions.

### Gene expression analysis

The raw data was normalised in the Affymetrix Expression Console Software using the robust multi-array average (RMA) method [12, 13]. Subsequent analysis of gene expression data was performed in the statistical computing language R (version 3.2.0) [14]. Tumour samples were categorised into *A3B*^del^ (samples with one- and two-copy *APOBEC3B* deletion) and *A3B*^wt^ (samples with no *APOBEC3B* deletion) breast cancers as previously described [9, 10]. For gene set enrichment analysis (GSEA; [http://www.broadinstitute.org/gsea/]), we used the list of all genes that were pre-ranked based on their moderated *t* statistic value using the ‘limma’ R package [15, 16]. Gene sets were defined by all gene ontology (GO) [17] terms extracted from class c5 in MsigDB [18]. A gene set was considered significantly enriched if its false discovery rate (FDR) *q* value was below 0.05.

### Data accession code

Microarray data are available from the Gene Expression Omnibus (GEO) database (accession number: GSE73540). The submission conformed to requirements for minimum information about a microarray experiment (MIAME) [19].

### Statistical analyses

The association between odds for breast cancer and *APOBEC3B* copy number was modelled using logistic regression (implemented in the Statistical Package for the Social Sciences (SPSS) version 16.0, SPSS Inc., Chicago, IL, USA). In our logistic regression model, we controlled for the effects of age, number of live births, age of first live birth, number of first-degree relatives with breast cancer, and oral contraceptive use. Odds ratios were estimated for one-copy and two-copy deletion genotypes compared with no deletion genotypes in the three ethnic groups. Odds ratio trend across genotypes was tested using the likelihood ratio test. The differences in the subject characteristics were determined using *t* test and chi-squared test while the differences in clinicopathologic features across *A3B*^del/del^, *A3B*^del/wt^ and *A3B*^wt/wt^ patients were tested using analysis of variance (ANOVA) and chi-squared test. The APOBEC family member gene expression across *A3B*^del/del^, *A3B*^del/wt^ and *A3B*^wt/wt^ breast cancers were compared using the Kruskal-Wallis test while immune scores and the relative frequency of tumour-infiltrating immune cell subsets across *A3B*^del/del^, *A3B*^del/wt^ and *A3B*^wt/wt^ breast cancers were compared using the Kruskal-Wallis and Mann-Whitney *U* test for three- and two-group comparison, respectively.

### TCGA and the METABRIC dataset

The two largest publicly available breast cancer datasets with both genomic and transcriptomic data, namely TCGA [20] and the Molecular Taxonomy of Breast Cancer International Consortium (METABRIC) [21] were analysed. For the TCGA breast cancer dataset, clinical information was retrieved from the cBio Cancer Genomics Portal [22] and germline *APOBEC3B* copy number status was obtained from Nik-Zainal et al. [9] while normalised RNASeqV2 gene expression profiles were retrieved from TCGA data portal. Gene expression values were transformed as X = log2 (X + 1), where X represents the normalised fragments per kilobase transcript per million mapped reads values. Altogether, 706 breast cancers from TCGA with normalised gene expression and germline *APOBEC3B* copy number status were collected and analysed. For the METABRIC dataset, clinical information was retrieved from the Synapse Commons archive (syn2133322; https://www.synapse.org/) and germline *APOBEC3B* copy number status was obtained from Cescon et al. [10] while normalised gene expression profiles were retrieved from the European genome-phenome archive (EGAS00000000083). Altogether, 1988 breast cancers from METABRIC with normalised gene expression profile and germline *APOBEC3B* copy number status were collected and analysed.

### Characterisation of tumour-infiltrating immune cells

Immune scores for breast cancers from TCGA and METABRIC datasets were defined using the Estimation of STromal and Immune cell in MAlignant Tumour (ESTIMATE) tissues using Expression data algorithm [23]. Cell type Identification By Estimating Relative Subsets Of known RNA Transcripts (CIBERSORT), a systematic and comprehensive tool to enumerate leukocyte abundance in bulk tumours using expression data from microarray experiment, was used to characterise the relative frequency of 22 tumour-infiltrating immune cell subtypes in breast cancers from METABRIC [24].

## Results

### Germline *APOBEC3B* deletion is associated with breast cancer risk but not clinicopathologic features of breast cancer in Malaysian women

The characteristics of the study cohort are shown in Table 1 and Additional file 1: Table S2. Both cases and controls had similar distribution of age, age at menarche, age at menopause and proportion receiving hormone replacement therapy. Breast cancer patients had significantly fewer live births (*P* < 0.01), and were younger at age of first live birth (*P* < 0.01). The fewer number of live births in cases was consistent with other epidemiological studies on breast cancer risk but the younger age of first live birth in cases was not consistent with other studies. One possible explanation is that our opportunistic mammography screening programme attracted women with higher socioeconomic status compared to our cases, which was a hospital-based cohort of patients. As this variable is unlikely to impact significantly on the genetic association, we proceeded with the genotype analysis [25]. Indeed, the genotype distributions of *APOBEC3B* in all three ethnic groups were in good agreement with expectations under Hardy-Weinberg equilibrium (Additional file 1: Table S3).

**Table 1:** Demographic characteristics and known breast cancer risk factors of study participants^a^

Category	Cases (*N* = 1451)	Controls (*N* = 1442)	*P* value
Demographic factors			
Age (year)	51.6 ± 7.5	51.4 ± 7.3	0.49^b^
Reproductive risk factors			
Age at menarche (year)	13.0 ± 1.5	12.9 ± 1.4	0.05^b^
Age at menopause (year)^d^	49.4 ± 4.5	49.3 ± 4.4	0.54^b^
Number of live births^e^	2.9 ± 1.3	3.2 ± 1.5	<0.01^b^
Age at first live birth (year)^e^	26.4 ± 5.0	27.5 ± 4.9	<0.01^b^
Other risk factors			
First-degree relatives with breast cancer (%)	13.2	11.0	0.08^c^
First- or second-degree relatives with breast cancer (%)	19.6	17.8	0.23^c^
Oral contraceptive (%)^f^	28.2	31.5	0.06^c^
Hormone replacement therapy (%)^f^	9.5	9.8	0.80^c^

^a^Unless otherwise specified, data are presented in mean ± standard deviation

^b^ *t* test

^c^Chi-squared test

^d^Among postmenopausal women

^e^Among parous women

^f^Ever user

The estimated minor allelic frequency (± standard error) of *APOBEC3B* in females of Chinese, Malay and Indian descent were 0.35 ± 0.01, 0.42 ± 0.02 and 0.16 ± 0.02, respectively. We found germline *APOBEC3B* deletion was associated with breast cancer risk, with ORs of 1.23 [95 % Cl: 1.05, 1.44] for one-copy deletion and 1.38 [95 % Cl: 1.10, 1.74] for two-copy deletion (*P* = 0.005; Table 2) compared to women with no deletion. After adjusting for age, number of live births and age of first live birth, there was negligible change in our results although number of live births and age of first live birth were significant in the logistic regression model (*P* < 0.05; data not shown). The adjusted odds ratio were 1.26 (95 % Cl: 1.05, 1.52]) for one-copy deletion and 1.36 (95 % Cl: [1.05, 1.78]) for two-copy deletion compared with women with no deletion (*P* = 0.014; data not shown). The association was also observed after stratifying the study group by ethnicity, but it was only statistically significant in Chinese women (Additional file 1: Table S4).

**Table 2:** Association between germline *APOBEC3B* deletion and breast cancer risk in Malaysian women

Genotype	Cases (*N* = 1451) *n* (%)	Controls (*N* = 1442) *n* (%)	OR (95 % Cl)^a^
*A3B* ^wt/wt^	591 (40.7)	670 (46.5)	1.00 (reference)
*A3B* ^del/wt^	649 (44.7)	599 (41.5)	1.23 [1.05, 1.44]
*A3B* ^del/del^	211 (14.5)	173 (12.0)	1.38 [1.10, 1.74]
*P*_trend_^b^			0.005

*A3B* ^*wt/wt*^ no deletion, *A3B* ^*del/wt*^ one-copy deletion, *A3B* ^*del/del*^ two-copy deletion

^a^Adjusted for age

^b^Test for trends of odds were two-sided and based on likelihood ratio tests

We observed no significant association between germline *APOBEC3B* deletion with any clinicopathologic features of breast cancers such as age of diagnosis, tumour grade, tumour size, lymph node involvement, estrogen receptor (ER) status, progesterone receptor (PR) status, human epithelial receptor 2 (HER2) status, and triple-negative breast cancer (TNBC) status in the overall cohort as well as in the Chinese and Indians (Table 3, Additional file 1: Table S5). However, we did observe HER2 status to be significantly associated with non-carriers of *APOBEC3B* deletion in the Malays with frequency of 61.9 % compared to one- and two-copy *APOBE3B* deletion carriers with frequency of 39.1 % and 36.2 %, respectively (*P* = 0.01; Additional file 1: Table S5).

**Table 3:** Association between *APOBEC3B* copy number and clinicopathologic features of breast cancers^a^

Clinical variables	Data available *N* (%)	*A3B* ^wt/wt^	*A3B* ^del/wt^	*A3B* ^del/del^	*P* value
Age (year)	1451 (100)	50.0 ± 7.4	51.0 ± 7.8	51.0 ± 7.1	0.23^b^
Grade 1/2/3 (%)	1140 (78.6)	14/51/36	11/54/36	14/51/36	0.76^c^
Size (cm)	1220 (84.1)	2.5 ± 2.5	2.5 ± 2.2	2.5 ± 1.7	0.93^b^
Node + (%)	1308 (90.1)	47.7	44.4	39.7	0.17^c^
ER+ (%)	1370 (94.4)	65.8	68.7	65.2	0.49^c^
PR+ (%)	1346 (92.8)	51.1	51.0	47.5	0.65^c^
HER2 (%)	1328 (91.5)	49.0	44.6	40.4	0.09^c^
TNBC (%)	1240 (87.7)	12.5	12.2	17.5	0.17^c^

*A3B* ^*wt/wt*^ no deletion, *A3B* ^*del/wt*^ one-copy deletion, *A3B* ^*del/del*^ two-copy deletion, *ER+* estrogen receptor positive, *PR+* progesterone receptor positive, *HER2* human epithelial receptor 2*, TNBC* triple-negative breast cancer

^a^Unless otherwise specified, data are presented in median ± interquartile range

^b^ANOVA

^c^Chi-squared test

### Germline *APOBEC3B* deletion is associated with *APOBEC3B* expression but not with other *APOBEC* family members

**Fig. 1:**
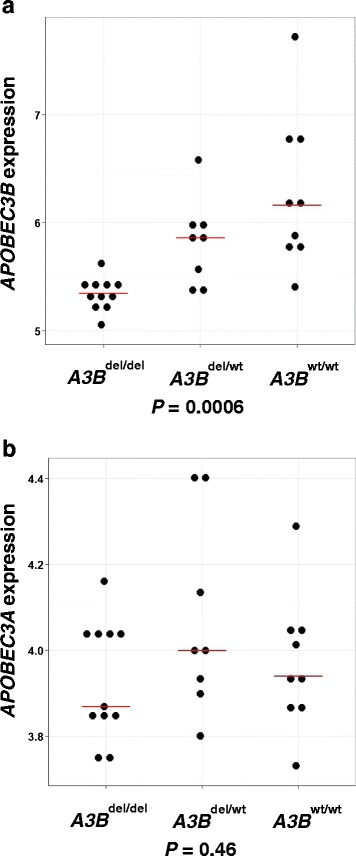
The relationship between germline *APOBEC3B* deletion status and expression of (**a**) *APOBEC3B* and (**b**) *APOBEC3A. Horizontal bars* represent median values. *P* values derived from Kruskal-Wallis test. *A3B* ^del/del^ two-copy deletion, *A3B* ^del/wt^ one-copy deletion, *A3B* ^wt/wt^ no deletion, *APOBEC3A* apolipoprotein B mRNA-editing enzyme, catalytic polypeptide-like 3A, *APOBEC3B* apolipoprotein B mRNA-editing enzyme, catalytic polypeptide-like 3B

We compared the gene expression of the *APOBEC* family members in individuals with different *APOBEC3B* copy number. We found *APOBEC3B* expression to be significantly associated with *APOBEC3B* copy number (*P* < 0.001), but *APOBEC3A* expression was not observed to be significantly higher in *APOBEC3B* deletion carriers (Fig. 1). There was also no association between *APOBEC3B* copy number and expression of other *APOBEC* family members (Additional file 1: Figure S1). The expression of apolipoprotein B mRNA-editing enzyme, catalytic polypeptide-like 4 (*APOBEC4*) was observed to be only marginally significant across *APOBEC3B* copy number (*P* = 0.04), and its expression was not found to be associated with the *APOBEC3B* gene copy number.

### Breast cancers arising from *APOBEC3B* deletion carriers are enriched for immune response-related gene sets

We performed gene expression analysis to investigate the biological processes underlying the loss of *APOBEC3B* in our tumour samples. The result of the GSEA showed that *A3B*^del^ breast cancers were significantly enriched for immune response-related gene sets (FDR *q* value < 0.05; Table 4).

**Table 4:** Gene set enrichment analysis of *A3B* ^del^ vs. *A3B* ^wt^ breast cancers

Gene set	Normalised ES	FDR *q* value
Chemokine receptor binding	−1.97	0.02
Chemokine activity	−1.93	0.03
Inflammatory response	−1.86	0.04

*ES* enrichment score, *FDR* false discovery rate

### Loss of *APOBEC3B* deletion is associated with tumour-infiltrating immune cells

**Fig. 2:**
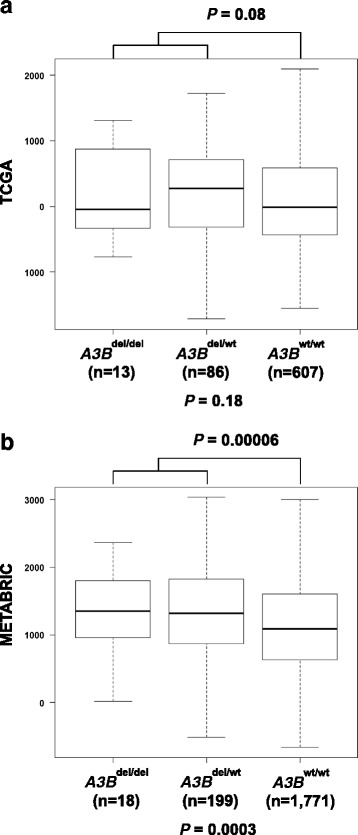
The relationship between germline *APOBEC3B* deletion status and immune scores in breast cancers from (**a**) TCGA and (**b**) METABRIC. Y-axis represents the immune score. *P* values for three-group comparison derived from Kruskal-Wallis test. *P* values from two-group comparison derived from Mann-Whitney *U* test. *A3B* ^del/del^ two-copy deletion, *A3B* ^del/wt^ one-copy deletion,* A3B* ^wt/wt^ no deletion, *METABRIC* Molecular Taxonomy of Breast Cancer International Consortium, *TCGA* The Cancer Genome Atlas

To investigate whether the enrichment of immune response-related gene sets in *A3B*^del^ breast cancers is associated with the presentation of tumour-infiltrating immune cells, we characterised the immune scores of *A3B*^del/del^, *A3B*^del/wt^ and *A3B*^wt/wt^ breast cancers from TCGA and METABRIC datasets using the ESTIMATE algorithm. We observed the immune scores to be significantly different across *APOBEC3B* copy number with *A3B*^del/del^, *A3B*^del/wt^, and *A3B*^wt/wt^ breast cancers with median immune scores of 1354, 1321 and 1093 (*P* < 0.001), respectively in breast cancers from METABRIC. However, we did not observe the immune scores to be significantly different across *APOBEC3B* copy number in the smaller sample set of breast cancers from TCGA with *A3B*^del/del^, *A3B*^del/wt^, and *A3B*^wt/wt^ breast cancers with median immune scores of −45, 273 and −13, respectively (Fig. 2). We extended our analysis by stratifying the breast cancers using PAM50 classification and found immune scores in luminal A and basal-like breast cancers from METABRIC to be significantly different across *APOBEC3B* copy number (*P* = 0.02 and *P* = 0.01, respectively) but not in luminal B and HER2-enriched breast cancers nor in any of the subtypes of breast cancers from TCGA (Additional file 1: Figures S2 and S3).

Using CIBERSORT, a bioinformatics tool used to infer immune cell composition from microarray datasets, we computed the relative frequency of 22 tumour-infiltrating immune cell subsets in breast cancers from METABRIC using the gene expression signatures. We observed monocytes and macrophages, CD8 T cells, mast cells, plasma cells, and CD4 T cells to be the most common immune cell subsets with mean fractions of 0.107, 0.080, 0.065, 0.047 and 0.039 respectively. Notably, breast cancers with at least one-copy *APOBEC3B* deletion had lower fraction of macrophages M2 compared to *A3B*^wt/wt^ breast cancers with mean fractions of 0.196 and 0.207, respectively, but this difference was only marginally significant (*P* = 0.04; Additional file 1: Table S6). Three-group comparison across *APOBEC3B* copy number for macrophages M2 fractions was not significant with *A3B*^del/del^, *A3B*^del/wt^, and *A3B*^wt/wt^ breast cancers with mean fractions of 0.179, 0.197 and 0.207, respectively (Additional file 1: Table S6).

## Discussion

In this study, we showed that germline *APOBEC3B* deletion is associated with an increased risk to breast cancer among Chinese, Malay and Indian women in Malaysia. Furthermore, we report that germline *APOBEC3B* deletion appears to be associated with an enrichment of immune response genes and that this may arise from the enrichment and activation of tumour-infiltrating immune cells.

Our breast cancer association results are consistent with previous findings in East Asian [7] and Caucasian women [8], which implicate germline *APOBEC3B* deletion as a susceptibility factor for breast cancer by conferring carriers a modest risk to the disease. Given the role of APOBEC3B as an endogenous mutator [5], it has been proposed that loss of APOBEC3B may be associated with less aggressive tumour phenotype [10]. However, we did not observe any association between germline *APOBEC3B* status and the clinicopathologic features of breast cancer patients. Our findings are consistent with that of a previous study analysing TCGA and the METABRIC datasets, which predominantly comprise patients of European descent [10].

Our gene expression analysis of fresh frozen tumour samples showed that in germline *APOBEC3B* deletion carriers, there is corresponding loss of gene expression of the *APOBEC3B* and this is consistent with previous studies [9, 10]. It has previously been proposed using transient transfection assays in 293T lines, that the deletion of *APOBEC3B* generates a novel *APOBEC3A* transcript, which is fused to the *APOBEC3B* 3′ UTR, and could result in a stabilized *APOBEC3A* mRNA transcript that may be more active than in non-*APOBEC3B* deletion carriers [26]. In our study, we did not find any upregulation of *APOBEC3A* or any of the other *APOBEC* cytosine deaminase family members, suggesting that there is no detectable feedback loop at the mRNA level that results in co-regulation of any of the members of this family. These gene expression results are consistent with data from TCGA, which similarly examined fresh frozen breast tumour samples but using a different analysis platform (transcriptomic sequencing) [9]. These data do not exclude the possibility that APOBEC3A may nonetheless be the endogenous source of mutations in *APOBEC3B* deletion carriers [27].

We showed that *A3B*^del^ breast cancers from our Asian cohort were enriched for immune response-related genes, indicating a possible involvement of immune response in carcinogenesis. This is consistent with previous results using TCGA and METABRIC datasets, where breast cancers arising from predominantly *APOBEC3B* deletion carriers of European descent were reported to be enriched for immune response-related gene sets [10]. Further characterisation of immune scores in breast cancers from the METABRIC dataset demonstrated that *A3B*^del/del^ and *A3B*^del/wt^ breast cancers present with significantly higher immune scores compared to *A3B*^wt/wt^, reflecting higher abundance of tumour-infiltrating immune cells in *A3B*^del^ breast cancers. Taken together, these data suggest that germline *APOBEC3B* deletion is associated with the enrichment of immune response-related gene sets arising from tumour-infiltrating immune cells. While it is anticipated that immune activation reflected by an increase in tumour-infiltrating immune cells would lead to reduced breast cancer risk in *APOBEC3B* deletion carriers, the conundrum between increased tumour-infiltrating immune cells and increased breast cancer risk in these individuals could be attributed to the increased propensity to develop higher mutation load in *APOBEC3B* deletion carriers [9] and this may override immune activation as previously suggested [10]. Although a number of factors are hitherto known to modulate immune cells infiltration, including history of pregnancy and lactation, commensal and pathobionts, and dietary and hormonal factors [28], to the best of our knowledge, this is the first time that germline genetic status has been suggested to modulate tumour-associated immune infiltrates.

## Conclusions

Taken together, we propose that tumour-infiltrating immune cells may be an important feature of breast cancers arising in women with *APOBEC3B* germline deletions and that this may be of particular interest in Asian women where the germline deletion is more common. Further research into the potential role of *APOBEC3B* genotype as a predictive biomarker for cancer prevention or anticancer immunotherapy may be warranted [10], particularly for Asian women.

## Abbreviations

AID, activation-induced cytidine deaminase; APOBEC1, apolipoprotein B mRNA-editing enzyme, catalytic polypeptide 1; APOBEC2, apolipoprotein B mRNA-editing enzyme, catalytic polypeptide-like 2; APOBEC3A, apolipoprotein B mRNA-editing enzyme, catalytic polypeptide-like 3A; APOBEC3B, apolipoprotein B mRNA-editing enzyme, catalytic polypeptide-like 3B; APOBEC3C, apolipoprotein B mRNA-editing enzyme, catalytic polypeptide-like 3C; APOBEC3D, apolipoprotein B mRNA-editing enzyme, catalytic polypeptide-like 3D; APOBEC3F, apolipoprotein B mRNA-editing enzyme, catalytic polypeptide-like 3F; APOBEC3G, apolipoprotein B mRNA-editing enzyme, catalytic polypeptide-like 3G; APOBEC3H, apolipoprotein B mRNA-editing enzyme, catalytic polypeptide-like 3H; APOBEC4, apolipoprotein B mRNA-editing enzyme, catalytic polypeptide-like 4; CIBERSORT, Cell type Identification By Estimating Relative Subsets Of known RNA Transcripts; Cl, confidence interval; ER, estrogen receptor; ESTIMATE, Estimation of STromal and Immune cell in MAlignant Tumour tissues using Expression data; FDR, false discovery rate; GO, gene ontology; GSEA, gene set enrichment analysis; HER2, human epithelial receptor 2; METABRIC, Molecular Taxonomy of Breast Cancer International Consortium; OR, odds ratio; TCGA, The Cancer Genome Atlas.

## Additional file

Additional file 1: Supplementary tables and figures. (DOCX 755 kb)
