# Supplementary material for: Germline APOBEC3B deletion is associated with breast cancer risk in an Asian multi-ethnic cohort and with immune cell presentation
Source: Breast Cancer Res. 2016 May 27;18:56. doi: 10.1186/s13058-016-0717-1 (PMC4884363; doi:10.1186/s13058-016-0717-1)
Supplement: Additional file 1: — Supplementary tables and figures. (DOCX 755 kb) [file 13058_2016_717_MOESM1_ESM.docx]

**Supplementary Tables and Figures**

**Table S1. Clinicopathologic features of breast tumours selected for microarray experiment.**

| **Sample** | **Genotype** | **Age**  **(year)** | **Stage** | **T** | **N** | **M** | **Grade** | **Size**  **(cm)** | **Site** | **Histology** |
| --- | --- | --- | --- | --- | --- | --- | --- | --- | --- | --- |
| 1  2  3  4  5  6  7  8  9  10  11  12  13  14  15  16  17  18  19  20  21  22  23  24  25  26  27  28 | *A3B*^wt/wt^  *A3B*^wt/wt^  *A3B*^wt/wt^  *A3B*^wt/wt^  *A3B*^wt/wt^  *A3B*^wt/wt^  *A3B*^wt/wt^  *A3B*^wt/wt^  *A3B*^wt/wt^  *A3B*^del/wt^  *A3B*^del/wt^  *A3B*^del/wt^  *A3B*^del/wt^  *A3B*^del/wt^  *A3B*^del/wt^  *A3B*^del/wt^  *A3B*^del/wt^  *A3B*^del/del^  *A3B*^del/del^  *A3B*^del/del^  *A3B*^del/del^  *A3B*^del/del^  *A3B*^del/del^  *A3B*^del/del^  *A3B*^del/del^  *A3B*^del/del^  *A3B*^del/del^  *A3B*^del/del^ | 42  56  62  45  65  50  41  56  62  54  69  41  45  64  45  65  46  64  64  68  66  55  63  47  44  51  47  56 | 2a  2a  3c  3a  2a  2a  1  2a  3a  2a  3c  1  2a  2a  2a  3b  2b  3b  4  3c  3c  3c  2a  2b  2a  2b  2a  2a | 1  2  2  2  2  2  1  1  2  2  2  1  2  2  2  2  2  1  3  4  2  4  1  2  1  2  2  1 | 1  0  3  2  0  0  0  1  2  0  3  0  0  0  0  2  1  2  3  3  3  2  1  1  1  1  0  1 | 0  0  0  0  0  0  0  0  0  0  0  0  0  0  0  0  0  0  1  0  0  0  0  0  0  0  0  0 | 1  2  3  2  2  2  2  2  3  2  3  3  3  2  2  2  2  3  2  2  2  2  2  2  2  2  3  2 | 1  2.6  7.6  2.5  2.2  2.5  0.6  2  4.5  2.5  4.3  1.2  2.8  3.4  4  5  5  2  3.5  4  4  2  1.5  2.3  1.2  2.5  3.5  2 | L  L  L  L  R  L  R  L  L  R  L  L  L  R  R  L  L  L  L  R  R  R  R  R  R  L  R  L | IDC  IDC  IDC  ILC  IDC  Mucinous  IDC  IDC  IDC  IDC  IDC  IDC  IDC  IDC  IDC  IDC  IDC  IDC  IDC  IDC  IDC  IDC  IDC  IDC  IDC  ILC  IDC  IDC |

L: Left; R: Right; IDC: Invasive ductal carcinoma; ILC: Invasive lobular carcinoma

**Table S2. Demographic characteristics and known breast cancer risk factors of study participants stratified by ethnicity ***

| **Category** | **Chinese** | | | **Indian** | | | **Malay** | | |
| --- | --- | --- | --- | --- | --- | --- | --- | --- | --- |
|  | **Cases**  **(N = 984)** | **Controls**  **(N = 985)** | ***P* value** | **Cases**  **(N = 245)** | **Controls**  **(N = 239)** | ***P* value** | **Cases**  **(N = 222)** | **Controls**  **(N = 218)** | ***P* value** |
| **Demographic factors**  Age (year)  **Reproductive risk factors**  Age at menarche (year)  Age at menopause (year) ^†^  Number of live birth (year) ^‡^  Age at first live birth (year) ^‡^  **Other risk factors**  First degree relatives with  breast cancer (%)  First or second degree relatives  with breast cancer (%)  Oral contraceptive ᵡ (%)  Hormone replacement therapy ᵡ (%) | 51.8 ± 7.3  13.0 ± 1.5  49.4 ± 4.6  2.8 ± 1.3  26.8 ± 5.1  14.3  21.3  29.5  10.1 | 51.8 ± 7.3  12.9 ± 1.4  49.5 ± 4.3  3.0 ± 1.3  27.9 ± 4.7  12.5  19.6  30.4  10.4 | 0.96 ^a^  0.07 ^a^  0.70 ^a^  <0.01 ^a^  <0.01 ^a^  0.24 ^b^  0.34 ^b^  0.69 ^b^  0.88 ^b^ | 53.0 ± 8.3  13.0 ± 1.3  49.4 ± 4.5  3.0 ± 1.3  25.8 ± 5.2  11.8  15.9  17.2  9.2 | 52.4 ± 7.4  12.9 ± 1.4  48.5 ± 5.0  3.4 ± 1.6  26.6 ± 5.7  10.5  15.1  23.8  10.9 | 0.36 ^a^  0.50 ^a^  0.17 ^a^  0.04 ^a^  0.11 ^a^  0.67 ^b^  0.80 ^b^  0.09 ^b^  0.65 ^b^ | 49.3 ± 7.3  12.9 ± 1.5  49.9 ± 3.9  3.3 ± 1.5  25.8 ± 4.4  9.9  15.8  34.9  7.1 | 48.9 ± 6.6  12.9 ± 1.3  49.3 ± 3.9  4.0 ± 1.7  26.3 ± 4.1  5.0  12.8  44.5  6.0 | 0.49 ^a^  0.66 ^a^  0.35 ^a^  <0.01 ^a^  0.27 ^a^  0.07 ^b^  0.42 ^b^  0.05 ^b^  0.70 ^b^ |

* Unless otherwise specified, data are presented in mean ± standard deviation

^a^ t-test

^b^ Chi-squared test

^†^ Among postmenopausal women

^‡^ Among parous women

ᵡ Ever user

**Table S3. Genotype frequencies and test for Hardy-Weinberg equilibrium of germline *APOBEC3B* deletion of controls.**

| **Ethnicity** | **Genotype** | **Observed**  **N (%)** | **Expected**  **N (%)** | ***P* value** |
| --- | --- | --- | --- | --- |
| Chinese  Indian  Malay | *A3B*^wt/wt^  *A3B*^del/wt^  *A3B*^del/del^  *A3B*^wt/wt^  *A3B*^del/wt^  *A3B*^del/del^  *A3B*^wt/wt^  *A3B*^del/wt^  *A3B*^del/del^ | 422 (42.8)  438 (44.5)  125 (12.7)  172 (72.0)  60 (25.1)  7 (2.9)  76 (34.9)  101 (46.3)  41 (18.8) | 416 (42.2)  448 (45.8)  121 (12.3)  173 (72.4)  61 (25.5)  5 (2.1)  73 (33.5)  106 (48.6)  39 (17.9) | 0.90  0.84  0.89 |

**Table S4. Association between germline *APOBEC3B* deletion and breast cancer risk in Malaysian women stratified by ethnicity.**

| **Ethnicity** | **Genotype** | **Cases**  **N (%)** | **Controls**  **N (%)** | **OR (95% Cl) †** |
| --- | --- | --- | --- | --- |
| Chinese  Indian  Malay | *A3B*^wt/wt^  *A3B*^del/wt^  *A3B*^del/del^  *P* _trend_ ^‡^  *A3B*^wt/wt^  *A3B*^del/wt^  *A3B*^del/del^  *P* _trend_ ^‡^  *A3B*^wt/wt^  *A3B*^del/wt^  *A3B*^del/del^  *P* _trend_ ^‡^ | 367 (37.3)  465 (47.3)  152 (15.4)  158 (64.5)  77 (31.4)  10 (4.1)  66 (29.7)  107 (48.2)  49 (22.1) | 422 (42.8)  438 (44.5)  125 (12.7)  172 (72.0)  60 (25.1)  7 (2.9)  76 (34.9)  101 (46.3)  41 (18.8) | 1.00 (reference)  1.22 [1.01,1.48]  1.40 [1.06,1.84]  0.026  1.00 (reference)  1.38 [0.93,2.07]  1.53 [0.57,4.13]  0.227  1.00 (reference)  1.22 [0.79,1.86]  1.37 [0.80,2.32]  0.478 |

^†^ Adjusted for age

^‡^ Likelihood ratio test for trends of odds ratio

**Table S5. Association between *APOBEC3B* copy number and clinicopathologic features of breast cancers stratified by ethnicity. ***

| **Ethnicity** | **Clinical variables** | **Data available**  **N (%)** | ***A3B*^wt/wt^** | ***A3B*^del/wt^** | ***A3B*^del/del^** | ***P* value** |
| --- | --- | --- | --- | --- | --- | --- |
| Chinese  Indian  Malay | Age (year)  Grade 1/2/3 (%)  Size (cm)  Node+ (%)  ER+ (%)  PR+ (%)  HER2+ (%)  TNBC (%)  Age (year)  Grade 1/2/3 (%)  Size (cm)  Node+ (%)  ER+ (%)  PR+ (%)  HER2 (%)  TNBC (%)  Age (year)  Grade 1/2/3 (%)  Size (cm)  Node+ (%)  ER+ (%)  PR+ (%)  HER2+ (%)  TNBC (%) | 984 (100)  776 (78.9)  831 (84.5)  877 (89.1)  927 (94.2)  909 (92.4)  898 (91.3)  858 (87.2)  245 (100)  189 (77.1)  209 (85.3)  230 (93.9)  232 (94.7)  228 (93.1)  228 (93.1)  220 (89.8)  222 (100)  175 (78.8)  180 (81.1)  201 (90.5)  211 (95.0)  209 (94.1)  202 (91.0)  195 (87.8) | 51.0 ± 7.2  13/53/34  2.5 ± 2.0  42.8  67.0  50.0  48.1  12.4  51.0 ± 7.8  18/44/38  3.0 ± 2.0  52.4  60.3  51.4  45.6  16.2  46.0 ± 6.8  9/55/36  2.1 ± 2.9  61.0  73.0  56.2  61.9  4.8 | 52.0 ± 7.5  11/55/35  2.5 ± 1.9  42.0  71.1  52.0  46.9  10.3  54.0 ± 8.5  10/49/41  3.0 ± 2.0  52.5  64.8  48.6  37.5  15.9  47.0 ± 7.8  14/50/58  3.0 ± 2.8  49.0  61.0  48.5  39.1  18.4 | 51.0 ± 6.8  14/49/38  2.5 ± 1.8  38.3  66.4  45.0  41.6  16.4  53.5 ± 11.4  11/56/33  3.0 ± 3.5  25  40.0  40.0  44.4  33.3  50.0 ± 6.7  15/55/30  2.5 ± 1.9  47.8  66.7  56.2  36.2  17.4 | 0.38 ^a^  0.78 ^b^  0.75 ^a^  0.67 ^b^  0.37 ^b^  0.36 ^b^  0.43 ^b^  0.17 ^b^  0.07 ^a^  0.67 ^b^  0.68 ^a^  0.14 ^b^  0.32 ^b^  0.75 ^b^  0.52 ^b^  0.40 ^b^  0.36 ^a^  0.85 ^b^  0.72 ^a^  0.27 ^b^  0.29 ^b^  0.53 ^b^  0.01 ^b^  0.05 ^b^ |

* Unless otherwise specified, data are presented in median ± interquartile range

^a^ ANOVA

^b^ Chi-squared test

**Table S6. Estimated fraction of tumor-infiltrating immune cell subsets in breast cancers from METABRIC***

| **Genotype** | **All (n = 1,988)** | ***A3B*^del/del^ (n = 18)** | ***A3B*^del/wt^ (n = 199)** | ***A3B*^del^ (n = 217)** | ***A3B*^wt^ (n = 1,771)** | ***P value* ^a^** | ***P value* ^b^** |
| --- | --- | --- | --- | --- | --- | --- | --- |
| **B cells** | 0.019 ± 0.030 | 0.014 ± 0.017 | 0.020 ± 0.027 | 0.020 ± 0.026 | 0.019 ± 0.030 | 0.21 | 0.12 |
| Naive B cells | 0.024 ± 0.030 | 0.019 ± 0.018 | 0.025 ± 0.027 | 0.025 ± 0.026 | 0.024 ± 0.030 | 0.21 | 0.10 |
| Memory B cells | 0.014 ± 0.029 | 0.009 ± 0.015 | 0.015 ± 0.026 | 0.014 ± 0.025 | 0.014 ± 0.030 | 0.78 | 0.71 |
| **Plasma cells** | 0.047 ± 0.054 | 0.053 ± 0.042 | 0.046 ± 0.057 | 0.047 ± 0.056 | 0.047 ± 0.054 | 0.42 | 0.73 |
| **CD8 T cells** | 0.080 ± 0.062 | 0.073 ± 0.057 | 0.084 ± 0.062 | 0.083 ± 0.061 | 0.079 ± 0.062 | 0.50 | 0.36 |
| **CD4 T cells** | 0.039 ± 0.053 | 0.037 ± 0.044 | 0.039 ± 0.050 | 0.039 ± 0.049 | 0.039 ± 0.053 | 0.66 | 0.37 |
| Naive CD4 T cells | 0.007 ± 0.024 | 0.006 ± 0.016 | 0.007 ± 0.020 | 0.007 ± 0.019 | 0.007 ± 0.025 | 0.45 | 0.22 |
| Resting memory CD4 T cells | 0.081 ± 0.073 | 0.061 ± 0.058 | 0.079 ± 0.068 | 0.078 ± 0.067 | 0.081 ± 0.074 | 0.56 | 0.98 |
| Activated memory CD4 T cells | 0.012 ± 0.025 | 0.015 ± 0.029 | 0.014 ± 0.026 | 0.014 ± 0.026 | 0.011 ± 0.025 | 0.19 | 0.07 |
| Follicular helper CD4 T cells | 0.074 ± 0.039 | 0.079 ± 0.031 | 0.072 ± 0.039 | 0.073 ± 0.038 | 0.074 ± 0.040 | 0.61 | 0.75 |
| Regulatory CD4 T cells | 0.022 ± 0.028 | 0.024 ± 0.025 | 0.021 ± 0.026 | 0.021 ± 0.026 | 0.022 ± 0.029 | 0.65 | 0.71 |
| **γδ T cells** | 0.027 ± 0.037 | 0.049 ± 0.052 | 0.029 ± 0.038 | 0.031 ± 0.040 | 0.027 ± 0.037 | 0.08 | 0.11 |
| **Natural killer cells** | 0.019 ± 0.026 | 0.022 ± 0.031 | 0.018 ± 0.025 | 0.018 ± 0.025 | 0.019 ± 0.026 | 0.74 | 0.49 |
| Resting natural killer cells | 0.010 ± 0.022 | 0.008 ± 0.019 | 0.008 ± 0.019 | 0.008 ± 0.019 | 0.010 ± 0.023 | 0.44 | 0.20 |
| Activated natural killer cells | 0.028 ± 0.026 | 0.035 ± 0.034 | 0.027 ± 0.026 | 0.028 ± 0.027 | 0.027 ± 0.026 | 0.69 | 0.98 |
| **Monocytes/Macrophages** | 0.107 ± 0.099 | 0.099 ± 0.087 | 0.106 ± 0.097 | 0.106 ± 0.096 | 0.107 ± 0.099 | 0.90 | 0.85 |
| Monocytes | 0.027 ± 0.031 | 0.018 ± 0.015 | 0.027 ± 0.028 | 0.026 ± 0.027 | 0.027 ± 0.032 | 0.56 | 0.67 |
| Macrophages M0 | 0.107 ± 0.098 | 0.100 ± 0.110 | 0.107 ± 0.097 | 0.107 ± 0.098 | 0.107 ± 0.098 | 0.90 | 0.95 |
| Macrophages M1 | 0.088 ± 0.049 | 0.098 ± 0.049 | 0.095 ± 0.054 | 0.095 ± 0.053 | 0.087 ± 0.049 | 0.14 | 0.06 |
| Macrophages M2 | 0.206 ± 0.097 | 0.179 ± 0.060 | 0.197 ± 0.098 | 0.196 ± 0.095 | 0.207 ± 0.097 | 0.12 | 0.04 |
| **Dendritic cells** | 0.007 ± 0.019 | 0.009 ± 0.020 | 0.007 ± 0.020 | 0.007 ± 0.020 | 0.007 ± 0.019 | 0.48 | 0.60 |
| Resting dendritic cells | 0.006 ± 0.014 | 0.015 ± 0.027 | 0.005 ± 0.012 | 0.006 ± 0.014 | 0.006 ± 0.014 | 0.06 | 0.37 |
| Activated dendritic cells | 0.008 ± 0.023 | 0.003 ± 0.007 | 0.008 ± 0.026 | 0.008 ± 0.025 | 0.008 ± 0.023 | 0.81 | 0.85 |
| **Mast cells** | 0.065 ± 0.091 | 0.078 ± 0.111 | 0.065 ± 0.093 | 0.066 ± 0.094 | 0.065 ± 0.091 | 0.96 | 0.92 |
| Resting mast cells | 0.129 ± 0.092 | 0.155 ± 0.113 | 0.128 ± 0.096 | 0.130 ± 0.097 | 0.128 ± 0.092 | 0.60 | 0.84 |
| Activated mast cells | 0.001 ± 0.009 | 0.001 ± 0.004 | 0.002 ± 0.013 | 0.002 ± 0.013 | 0.001 ± 0.009 | 0.95 | 0.79 |
| **Eosinophils** | 0 ± 0.001 | 0 | 0 | 0 | 0 ± 0.001 | 0.48 | 0.22 |
| **Neutrophils** | 0.003 ± 0.007 | 0 ± 0.001 | 0.003 ± 0.007 | 0.002 ± 0.007 | 0.003 ± 0.007 | 0.32 | 0.99 |

γδ T cells: gamma delta T cells; *A3B*^del/del^: Two-copy deletion; *A3B*^del/wt^: One-copy deletion; *A3B*^del^; One- and two-copy deletion; *A3B*^wt/wt^: No deletion.

* Unless otherwise specified, data are presented in mean ± standard deviation.

^a^ *P* values derived from Kruskal-Wallis test for comparison between *A3B*^del/del^, *A3B*^del/wt^, and *A3B*^wt/wt^.

^b^ *P* values derived from Mann-Whiney U test for comparison between *A3B*^del^ and *A3B*^wt/wt^.


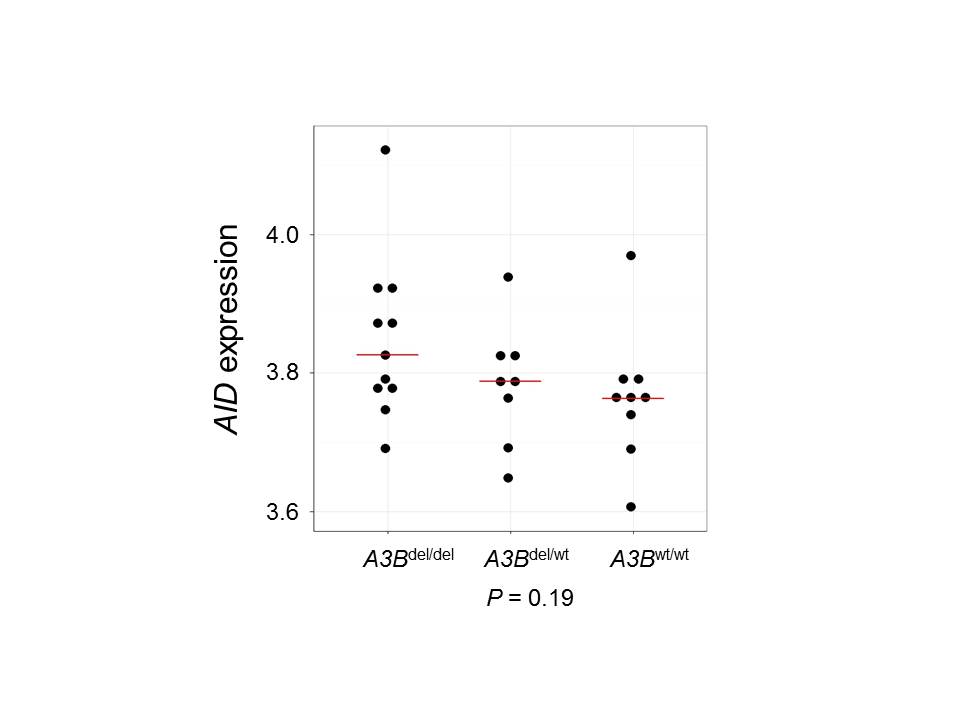

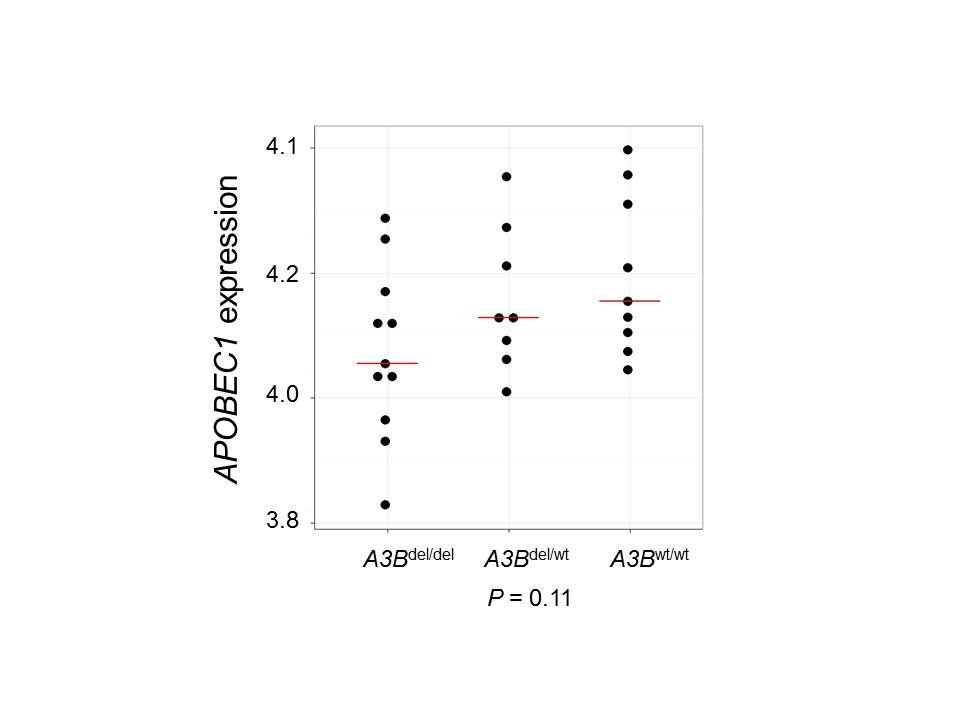

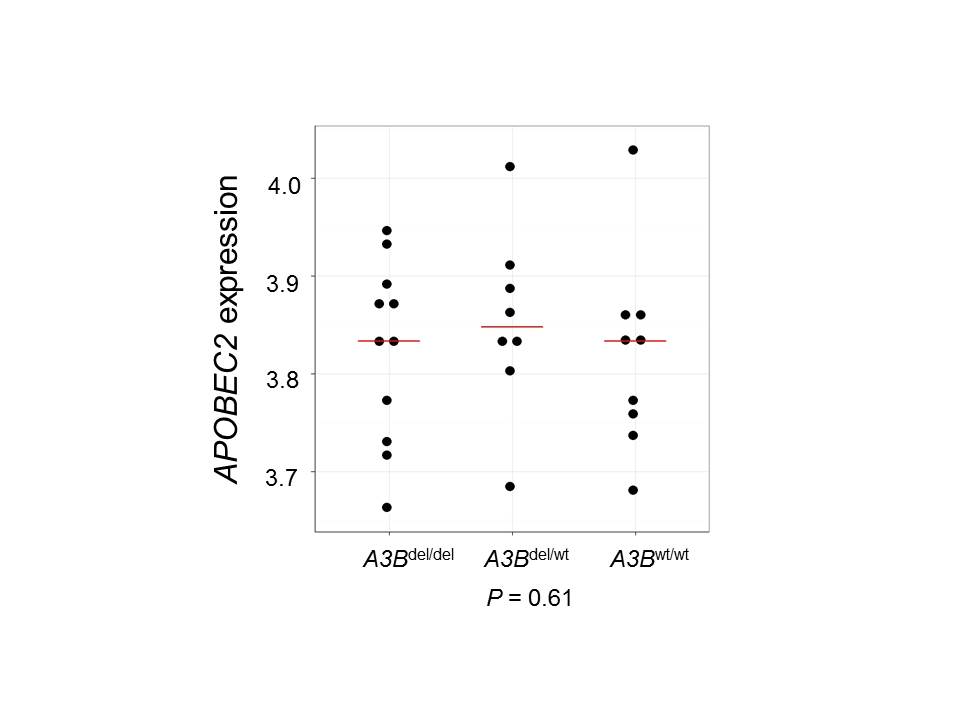


***P* = 0.61**

***P* = 0.11**

***P* = 0.19**


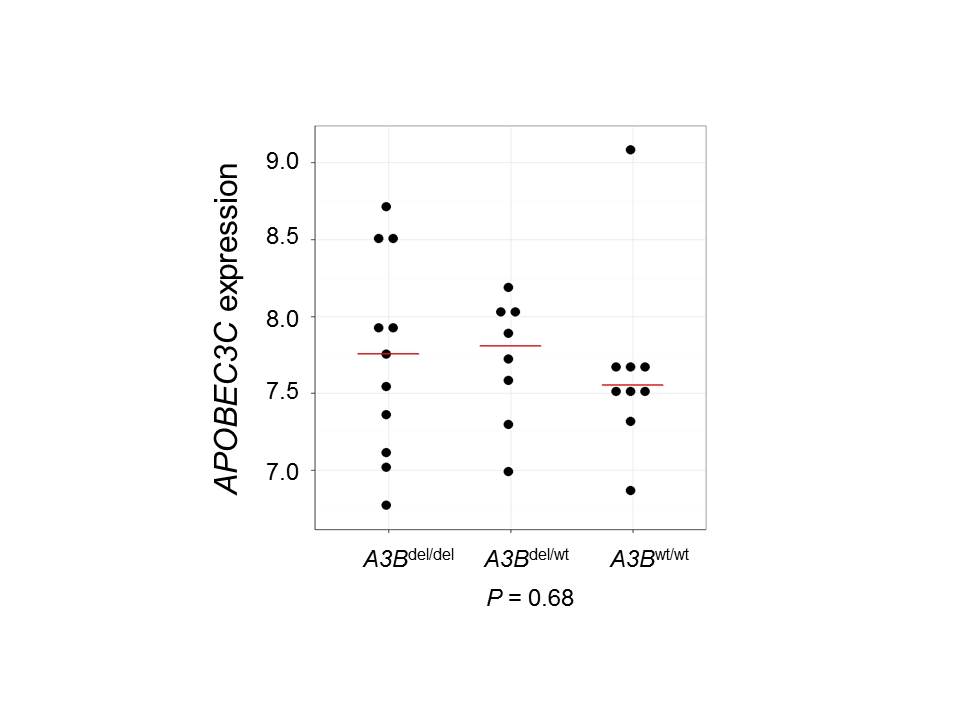

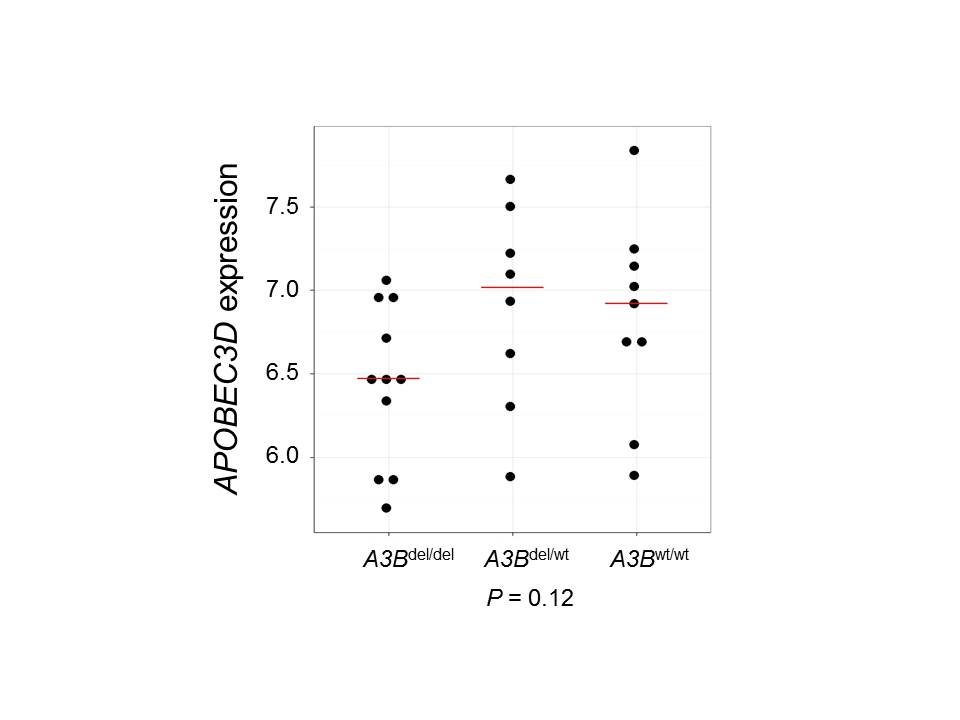

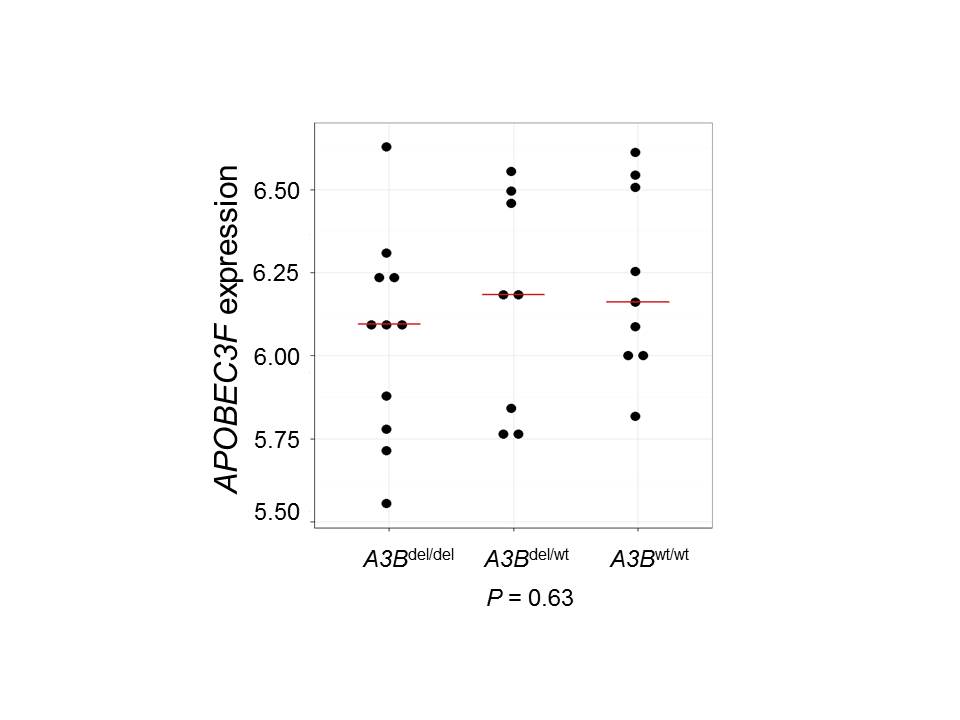


***P* = 0.63**

***P* = 0.12**

***P* = 0.38**


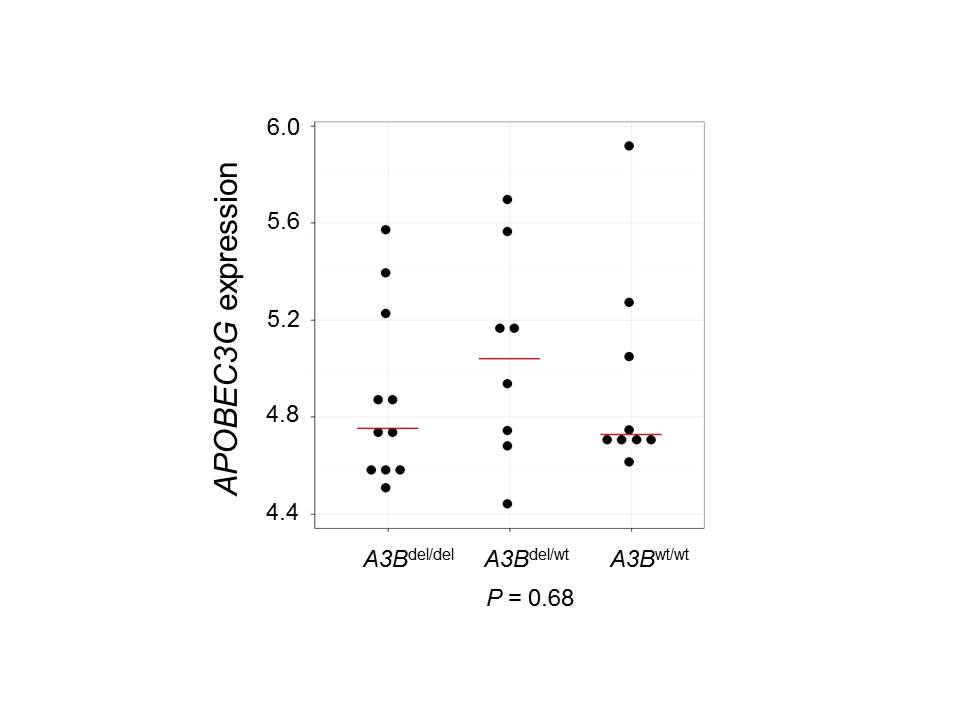

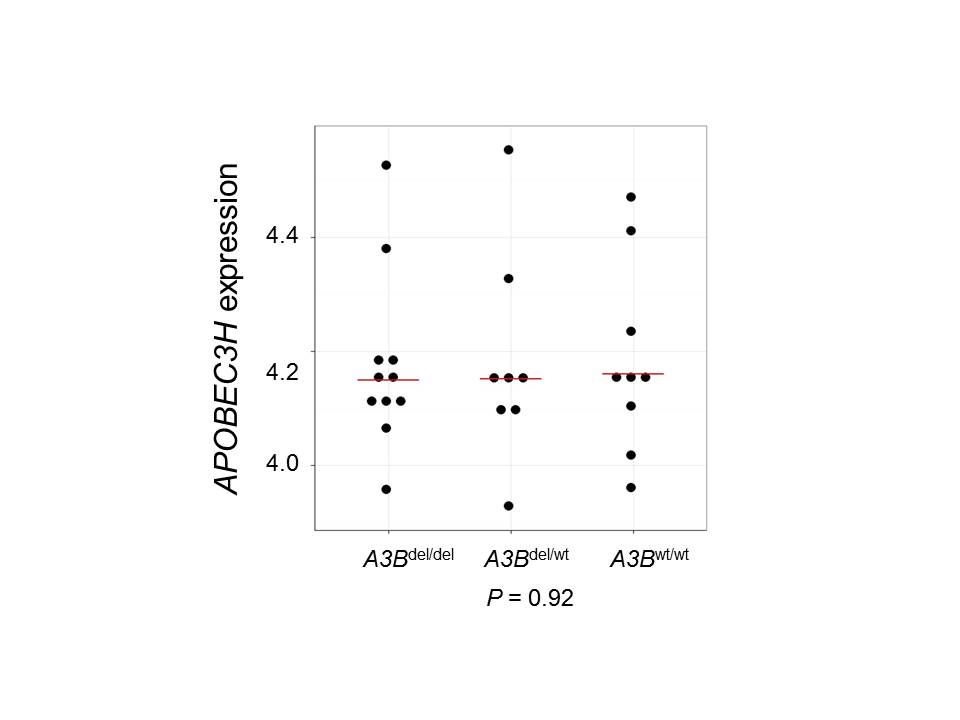

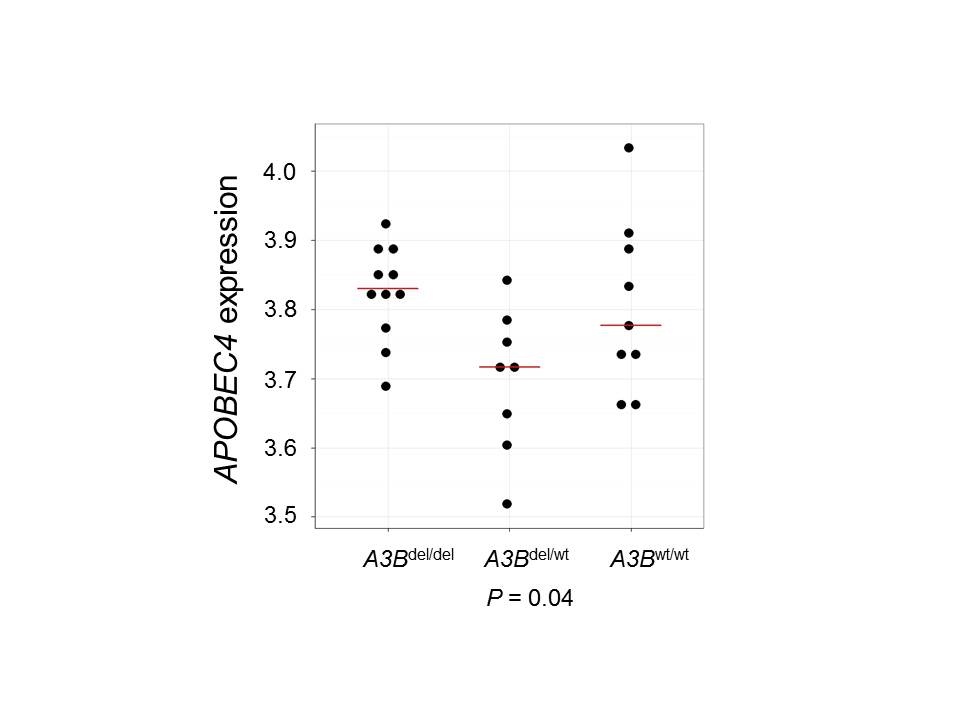


***P* = 0.04**

***P* = 0.92**

***P* = 0.68**

**Figure S1.** The relationship between germline *APOBEC3B* deletion status and expression levels of 9 different *APOBEC* family members**.** Horizontal bars represent median values. *P* values derived from Kruskal-Wallis test. *A3B*^del/del^: Two-copy deletion; *A3B*^del/wt^: One-copy deletion; *A3B*^wt/wt^: No deletion.


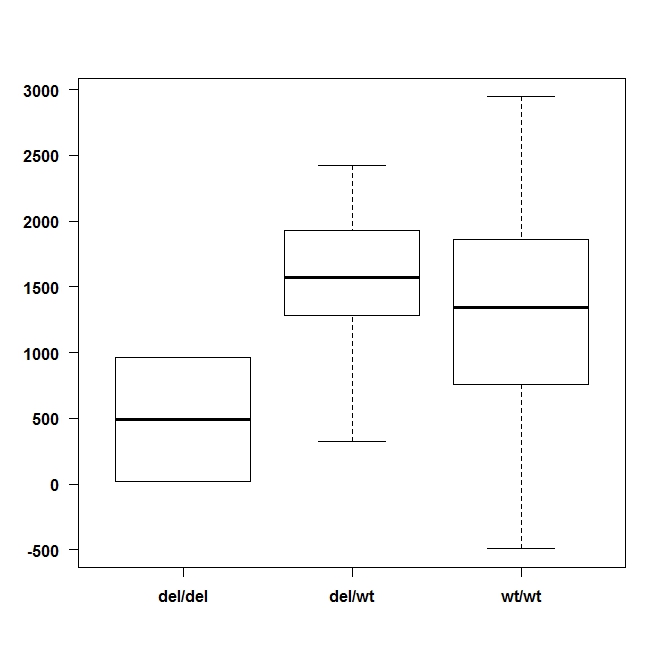

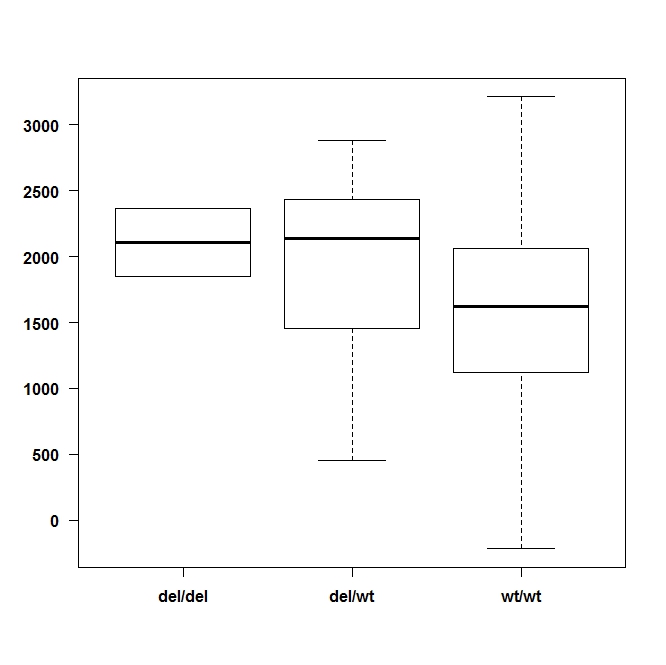

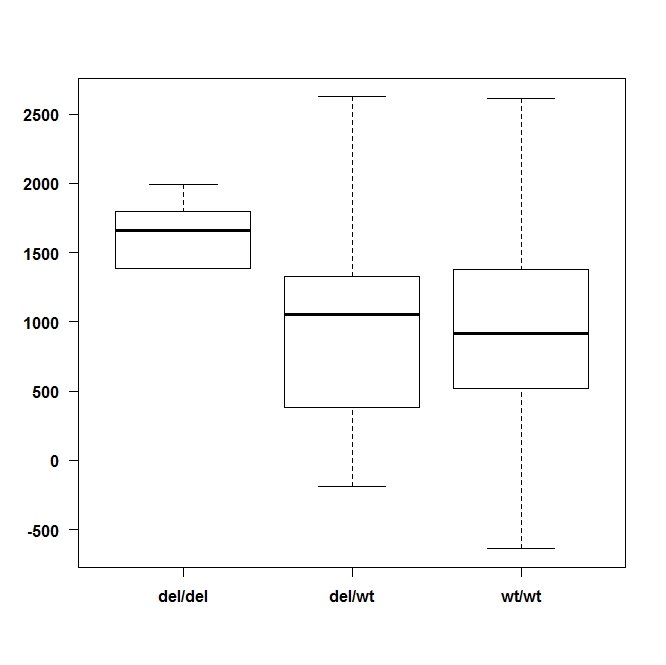

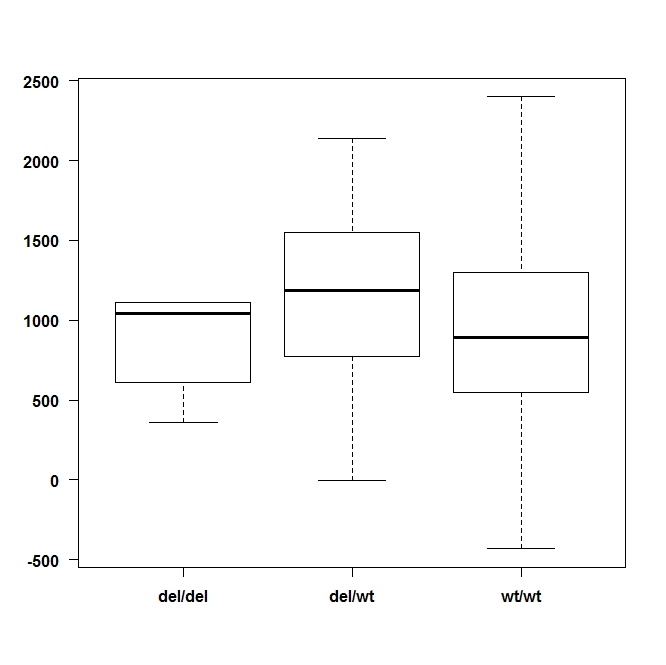


**d.**

**c.**

**b.**

**a.**

***P* = 0.07**

***A3B*^wt/wt^**

**(n=205)**

***A3B*^del/wt^**

**(n=31)**

***A3B*^del/del^**

**(n=2)**

***P* = 0.01**

***A3B*^wt/wt^**

**(n=290)**

***A3B*^del/wt^**

**(n=35)**

***A3B*^del/del^**

**(n=2)**

***P* = 0.14**

***A3B*^wt/wt^**

**(n=440)**

***A3B*^del/wt^**

**(n=44)**

***A3B*^del/del^**

**(n=5)**

***P* = 0.02**

***A3B*^wt/wt^**

**(n=649)**

***A3B*^del/wt^**

**(n=64)**

***A3B*^del/del^**

**(n=5)**

***P* = 0.61**

***P* = 0.003**

***P* = 0.005**

***P* = 0.46**

**HER2-enriched**

**Luminal B**

**Basal-like**

**Luminal A**

**Figure S2.** The relationship between germline *APOBEC3B* deletion status and immune scores in breast cancers from METABRIC after stratifying by PAM50 subtype. Y-axis represents the immune score. *P* values for 3-group comparison derived from Kruskal-Wallis test. *P* values from 2-group comparison derived from Mann-Whitney U test. *A3B*^del/del^: Two-copy deletion; *A3B*^del/wt^: One-copy deletion; *A3B*^wt/wt^: No deletion.


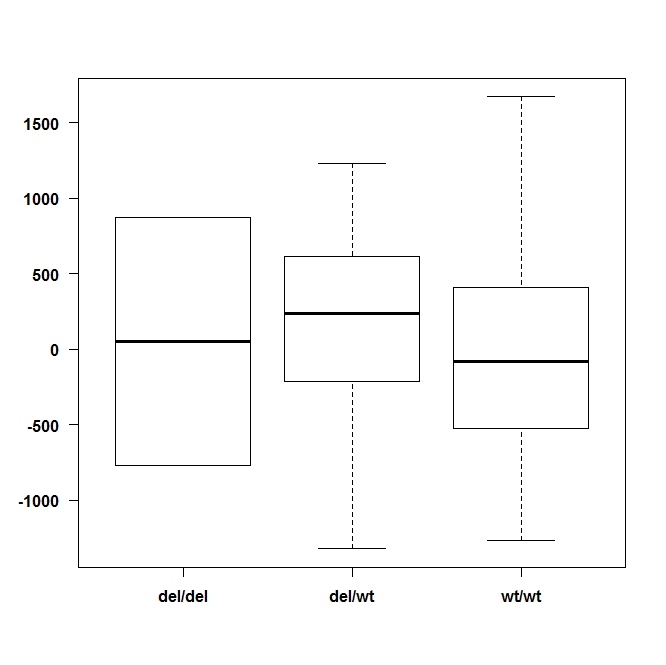

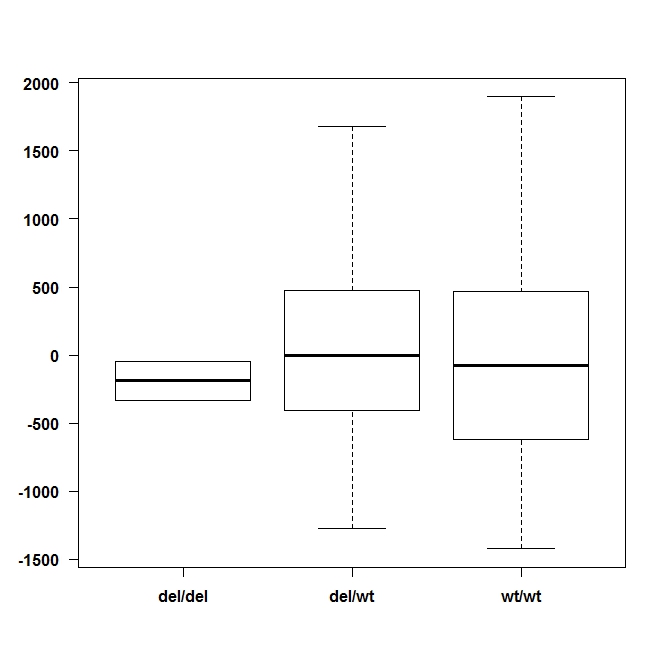

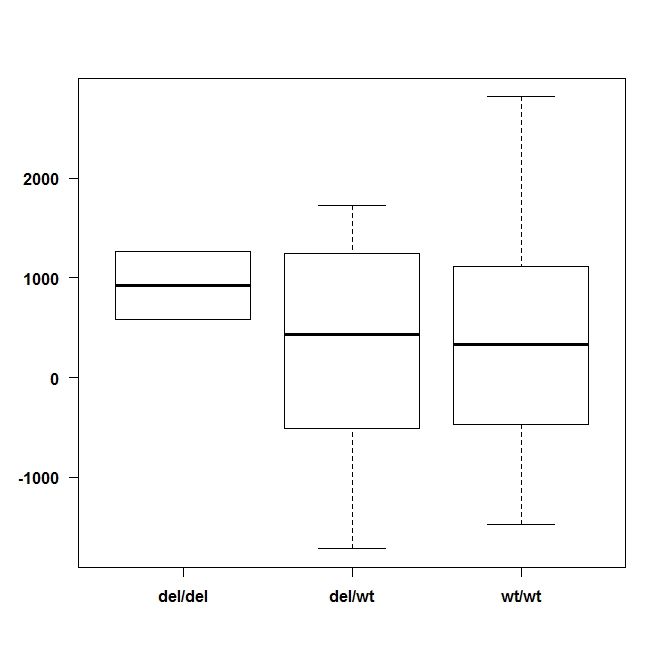

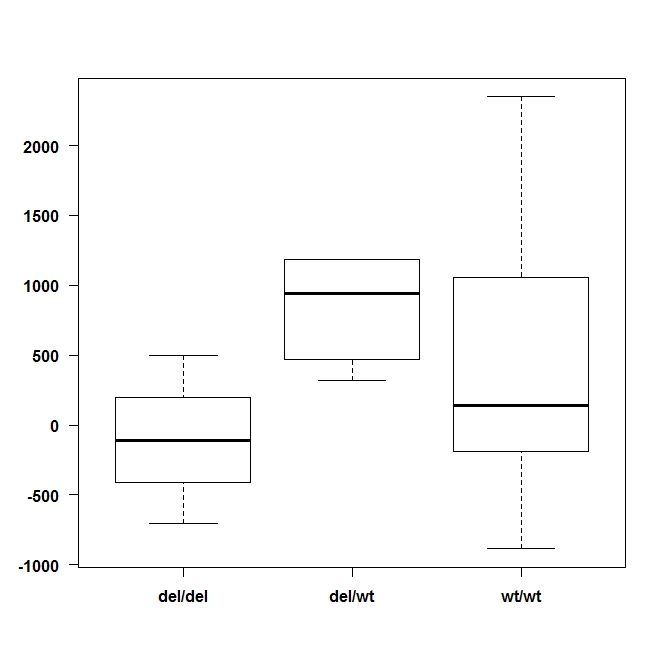


***P* = 0.61**

***P* = 0.43**

**Luminal B**

**HER2-enriched**

**Basal-like**

**Luminal A**

**a.**

**b.**

**c.**

**d.**

***P* = 0.74**

***P* = 0.13**

***A3B*^del/del^**

**(n=2)**

***A3B*^del/wt^**

**(n=29)**

***A3B*^wt/wt^**

**(n=170)**

***P* = 0.28**

***A3B*^del/del^**

**(n=2)**

***A3B*^del/wt^**

**(n=14)**

***A3B*^wt/wt^**

**(n=99)**

***P* = 0.82**

***A3B*^del/del^**

**(n=2)**

***A3B*^del/wt^**

**(n=12)**

***A3B*^wt/wt^**

**(n=71)**

***P* = 0.73**

***A3B*^del/del^**

**(n=3)**

***A3B*^del/wt^**

**(n=5)**

***A3B*^wt/wt^**

**(n=44)**

***P* = 0.13**

**Luminal A**

**Basal-like**

**Figure S3.** The relationship between germline *APOBEC3B* deletion status and immune scores in breast cancers from TCGA after stratifying by PAM50 subtype. Y-axis represents the immune score. *P* values for 3-group comparison derived from Kruskal-Wallis test. *P* values from 2-group comparison derived from Mann-Whitney U test. *A3B*^del/del^: Two-copy deletion; *A3B*^del/wt^: One-copy deletion; *A3B*^wt/wt^: No deletion.
